# Two ferroptosis-specific expressed genes NOX4 and PARP14 are considered as potential biomarkers for the diagnosis and treatment of diabetic retinopathy and atherosclerosis

**DOI:** 10.1186/s13098-024-01301-3

**Published:** 2024-03-05

**Authors:** Chen Li, QinHua Cai

**Affiliations:** Department of Ophthalmology, The First Affiliated Hospital of Soochow University, Shizi Street 188, Suzhou, 21006 Jiangsu China

**Keywords:** Diabetic retinopathy, Atherosclerosis, Ferroptosis, NOX4 and PARP14, Immune infiltration

## Abstract

**Objectives:**

Both Diabetic retinopathy (DR) and Atherosclerosis (AS) are common complications in patients with diabetes, and they share major pathophysiological similarities and have a common pathogenesis. Studies performed to date have demonstrated that ferroptosis plays a vital part in the occurrence and development of DR and AS, but its mechanism in the two diseases remains poorly understood.

**Methods:**

DR Chip data (GSE60436 and GSE102485) and AS chip data (GSE100927 and GSE57691) were obtained from the Gene Expression Omnibus (GEO) database. The screening of the differential expression genes (DEGs) was analyzed using the limma package, and the genes related to ferroptosis were obtained from the FerrDb V2 database. Two key genes (NOX4 and PARP14) were identified through external datasets validation and receiver operating characteristic (ROC) curve analysis. Gene Ontology (GO) and Gene Set Enrichment Analysis (GSEA) were used to conduct a functional enrichment analysis, and miRNA-mRNA networks were established. The CIBERSORT algorithm was applied to identify the immune cell infiltration between the disease group and control group. Next, the correlations between key genes and infiltrating immune cells were investigated by the Spearman method. Finally, the correlation between 2 key genes and ferroptosis markers was confirmed.

**Results:**

Nine ferroptosis differentially expressed genes (DE-FRGs) between DR and AS were identified in this study. NOX4 and PARP14 were selected as key genes for further analysis by external datasets and ROC curve analysis. The key genes NOX4, PARP14 and their correlated genes (such as CYBA, NOX1, NOX3, CYBB, PARP9, PARP10, and PARP15) are mainly enriched in oxidoreductase activity, protein ADP-ribosylation, superoxide metabolic process, reactive oxygen species metabolic process, PID pathway, and VEGFA-VEGFR2 pathway. A miRNA-mRNA network was constructed, and we got 12 miRNAs correlated with the target gene NOX4, 38 miRNAs correlated with the target gene PARP14. Three common miRNAs (hsa-miR-1-3p, hsa-miR-129-2-3p, and hsa-miR-155-5p) were observed in the network. Immune infiltration analysis displayed that activated B cell, MDSC, and Type 17 T helper cell are the common immune cells involved in the immune infiltration process of DR and AS. The results revealed that there are significant correlations between two key genes and most ferroptosis marker genes no matter in DR or AS.

**Conclusion:**

Ferroptosis-related genes NOX4 and PARP14 may be common biomarkers of DR and AS. Both were associated with immune infiltration in patients with DR and AS. Our data provide a theoretical basis for the early diagnosis and immunotherapy of the two diseases.

## Introduction

Diabetic retinopathy (DR) is the most common and devastating ocular complication in diabetes [1]. Atherosclerosis (AS) is also one of the common complications in diabetic patients. The association between DR and AS has attracted a large portion of research attention in recent years [2–4]. The American Diabetes Association (ADA) points out that DR is an independent predictor of type 2 diabetes (T2D) subclinical cardiovascular disease [5]. In T2D, DR alone or with other microvascular complications is associated with carotid atherosclerosis in patients [6, 7]. Furthermore, DR is associated with the occurrence of carotid atherosclerosis in type 1 diabetes mellitus (T1D) patients without history of cardiovascular disease. The presence of DR can determine the risk of carotid atherosclerotic in T1D patients [8]. The occurrence and development of DR and AS are very insidious and difficult to find. When vision loss or vascular damage has occurred, the best time for diagnosis and treatment is missed. Thus, this has major implications to further investigate the pathogenesis of DR and AS and identify new biomarkers for diagnosis.

Ferroptosis is an unconventional pattern of programmed cell death dependent on ferric ions. The morphology of ferroptosis is mitochondria shrank, condensed mitochondrial membrane density, mitochondrial crista reduction or disappearance [9]. The occurrence of ferroptosis and the inactivation of GPX4 lead to the increase of intracellular lipid reactive oxygen species (ROS), which attack lipids, induce lipid peroxidation, and leads to cell death [3]. Endothelial dysfunction caused by ROS is the initial link of AS occurrence [10]. Recent studies revealed that ferroptosis is highly linked with the pathogenesis of AS [11–13]. In addition, studies have also shown the function of ferroptosis in DR. The key characteristics of the occurrence and development of DR are the damage of retinal pigment epithelial (RPE) cells, which causes blood-retinal barrier disrupted, and the increase of the permeability of human retinal capillary endothelial cells (HRCECs). In DR, ferroptosis is involved in RPE cells and HRCECs death process [14, 15]. However, the pathogenesis, signaling pathways, and biomarkers of DR and AS related to ferroptosis remain unknown.

Bioinformatics methods enable us to understand the potential molecular mechanisms of diseases at a genetic level more deeply by screening molecules and displaying differences between patients and healthy individuals. This study aimed to identify hub genes associated with the pathogenesis of DR and AS. We obtained ferroptosis-related DEGs from the GEO database and FerrDb V2s database. Using integrated bioinformatics, enrichment analysis, and immune infiltration analysis, we identified common ferroptosis-related biomarkers for DR and AS and the associated immune cell infiltration status. These findings provide new insights into the molecular mechanisms involved in the pathophysiological processes of DR and AS.

## Materials and methods

### Microarray data retrieval

Four datasets (GSE60436, GSE100927, GSE102485, and GSE57691) related to DR and AS were downloaded from NCBI GEO (http://www.ncbi.nlm.nih.gov/geo) [16]. The GSE60436 (Illuminai HumanWG-6 v3.0 expression beadchip) was generated by the GPL6884 platform that included 6 fibrovascular membranes (FVMs) of retina samples from proliferative diabetic retinopathy (PDR) and 3 control samples [17]. The GSE100927 (Agilent-039494 SurePrint G3 Human GE v2 8 × 60 K Microarray 039381 (Probe Name version)) is acquired from the GPL17077 platform and contained 69 atherosclerotic lesions arteries samples and 35 control samples [18]. To better identify the key genes, two external datasets (GSE102485 and GSE57691) were used. The GSE102485 (Illumina NextSeq 500 (Homo sapiens)) is acquired from the GPL18573 platform and included 22 neovascular proliferative membrane specimens from patients with PDR and 3 control retina samples [19]. The GSE57691 (Illumina HumanHT-12 V4.0 expression beadchip) is generated by the GPL10558 platform comprising 9 atherosclerosis specimens obtained from aortic occlusive disease (AOD) patients and 10 control aortic specimens of organ donors [20].

### Acquisition of microarray data and identification of DE-FRGs

Microarray data were accessed from GEO using the “GEO query” R package. After data preprocessing, the normalized expression profile data were analyzed and DEGs were screened using the “Limma” package in R language, p < 0.05 and, | log2FC (Fold-change) |≥ 0.585 were used to define DEGs. The volcano plot and heatmap were created with R package “ggplot2” and “ComplexHeatmap” [21–23]. We used the FerrDb V2 database (http://www.zhounan.org/ferrdb) to acquire FRGs. Venn diagrams were created using the R package “VennDiagram” [24].

### Identification of key genes in DR and AS

Wilcoxon test was used for comparing differences between the disease group and the control group, and *p* < 0.05 suggests the difference was statistically significant. Receiver operating characteristic (ROC) curve analysis was generated with the “pROC” package in R [25], and the area under the curve (AUC) was calculated to assess the diagnostic value.

### miRNA-mRNA network construction

The miRNA target key genes prediction was performed by the TarBase V8.0 [26]. The mRNA-miRNA network was established and visualized through the Cytoscape (Version 3.7.1).

### Functional enrichment analysis

The functional association of target genes was analyzed using GeneMANIA (http://genemania.org). Gene Ontology (GO) and pathway enrichment analysis are achieved through the Metascape website (http://metascape.org/). Gene Set Enrichment Analysis (GSEA) associated with two key genes was performed using the “clusterProfiler” package, with gene set c2 (cp.v7.2.symbols.gmt) [27, 28].

### Analysis of immune infiltrating cells

The CIBERSORT method was adopted to investigate the immune cell infiltration between the disease group and the control group [29]. T-tests were used for immune infiltrating cell comparisons between the two groups. The Boxplot of immune cell proportions was visualized with the ggplot2 package (http://ggplot2.tidyverse.org). Correlation analysis between key genes and immune cells was identified using the Spearman correlation coefficient, and we visualized the results with lollipop plots.

### Statistical analysis

R software (version 3.6.3) and GraphPad Prism 5 were used for statistical analysis in this work. Data were expressed as mean ± standard deviation and unpaired Student’s t-test was employed for group comparisons. *p* < 0.05 suggests the difference was statistically significant.

## Results

### Identification of DE-FRGs in DR and AS

**Fig. 1:**
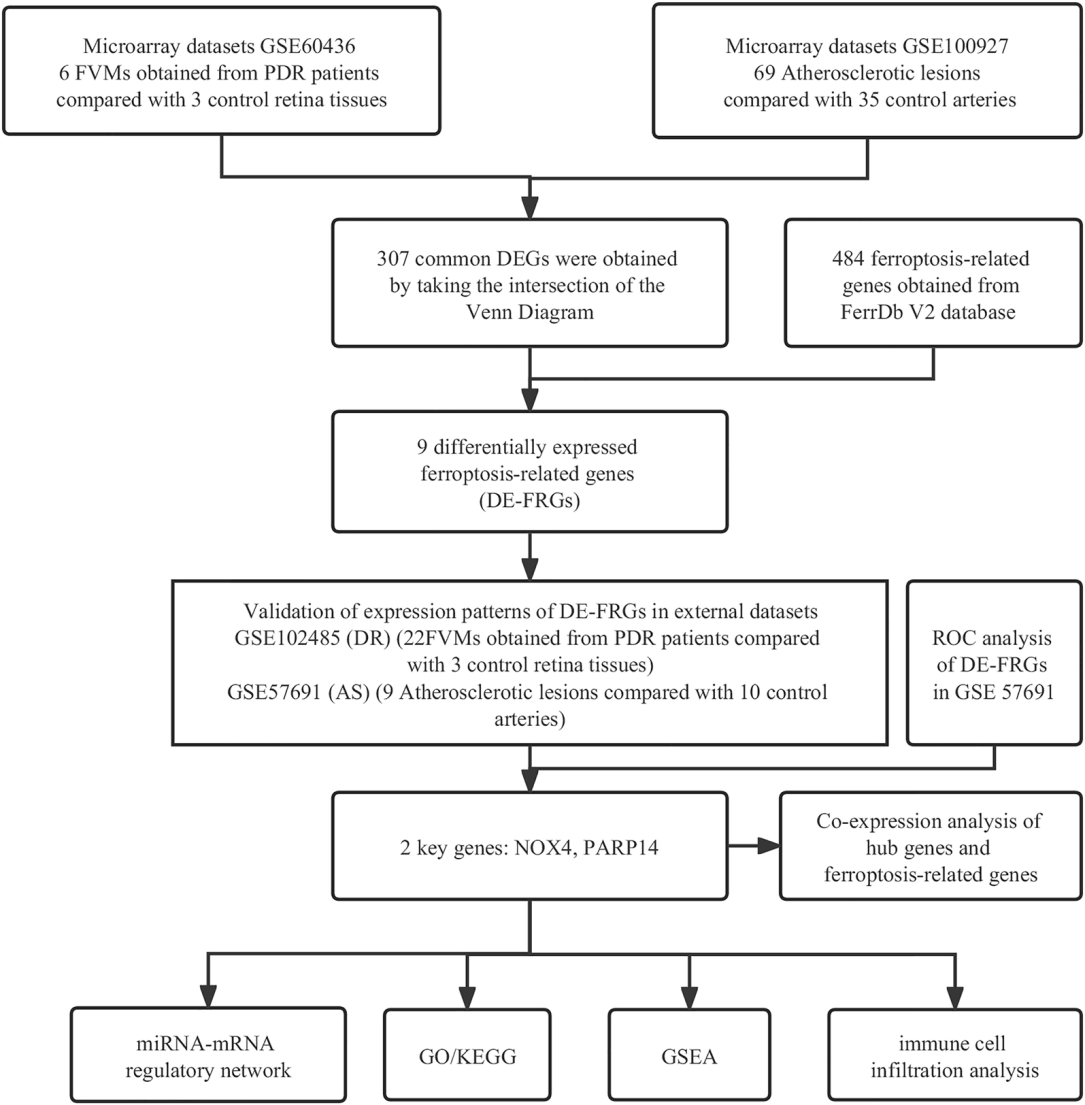
Flowchart of the bioinformatics analysis

**Fig. 2:**
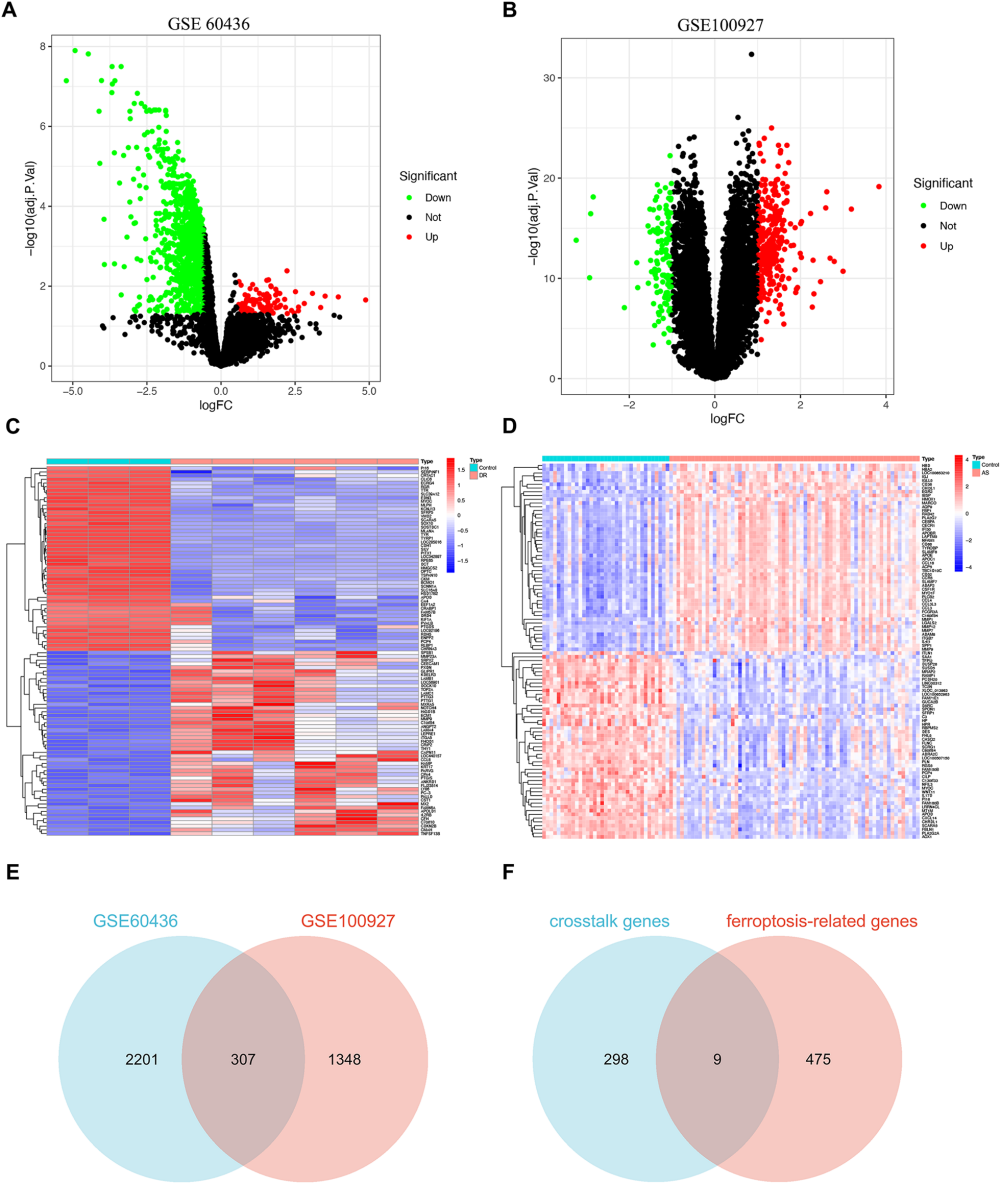
Identification of the DE-FRGs. **A**, **B** Volcano plot of DEGs in GSE60436 and in GSE100927, Red dots represent the upregulated, green dots denote the downregulated genes. **C**, **D** Heatmap of DEGs in GSE60436 and in GSE100927, Red bricks represent the more highly expressed DEGs, blue bricks denote lower expression DEGs. **E** Venn diagram displaying the crosstalk genes between GSE60436 and GSE100927. **F** Venn diagram displaying the DE-FRGs by taking the intersection of crosstalk genes and FRGs

Figure 1 illustrates the flowchart of dataset selection and the study design. 484 FRGs were acquired from the FerrDb V2 database, which was a database that commits to ferroptosis regulators and ferroptosis-related diseases association. The expressions of the DEGs in GSE60436 and GSE100927 were displayed in volcano plots and heatmaps (Fig. 2A–D). Through differential expression analysis of GSE60436 and GSE100927, 307 common genes were significantly differentially expressed in both DR and AS (Fig. 2E). We obtained 9 DE-FRGs including TLR4, PARP12, PARP14, CYBB, NOX4, ABCC5, BID, LIFR and SNCA by taking the intersection of DEGs and FRGs (Fig. 2F).

### Identification of the key genes in DR and AS

**Fig. 3:**
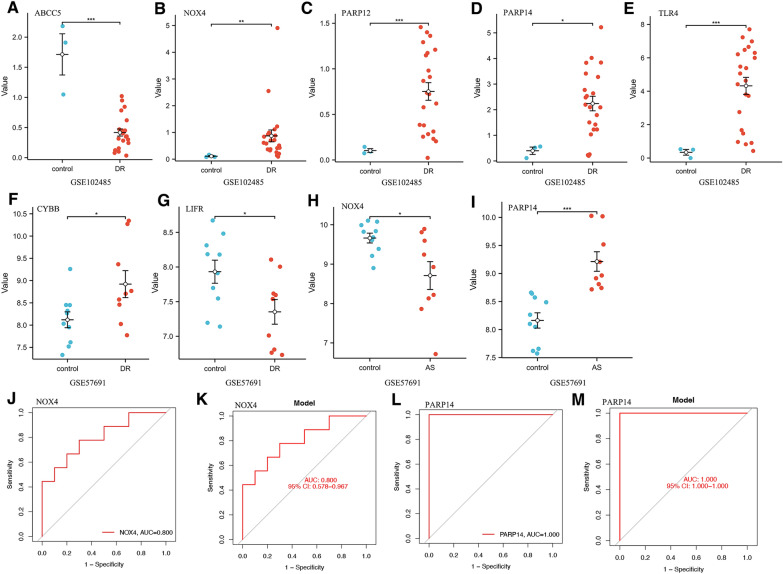
Verification of key genes for DR and AS. **A–E** The expressions of partial DE-FRGs in the GSE102485 dataset. **F–I** The expressions of partial DE-FRGs in the GSE57691 dataset. **J–M** ROC curve and AUC evaluated the diagnostic values of key genes in GSE57691 dataset. *p < 0.05, **p < 0.01, ***p < 0.001

To further identify the key genes and evaluate their diagnostic value, we observed the expressions of 9 DE-FRGs in two external validation datasets GSE102485 and GSE57691 to get robust diagnostic key genes. Among these genes, we found a significant difference in the expression of ABCC5, NOX4, PARP12, PARP14, and TLR4 in the DR group in comparison with the control in GSE102485 (Fig. 3A–E). In the dataset GES57691, the expression level of CYBB, LIFR, NOX4, and PARP14 showed a significant difference in the AS group when compared to controls (Fig. 3F–I). Of these, NOX4 and PARP14 showed a significant difference in the two disease groups compared with controls. The diagnostic accuracy of NOX4 and PARP14 in GSE57691 was assessed with ROC curves and AUC. It was found that both NOX4 and PARP14 had high diagnostic values with AUC = 0.80 and AUC = 1.00 (Fig. 3J–M). Collectively, NOX4 and PARP14 were considered key genes and were chosen for subsequent analysis.

### Construction of the miRNA-mRNA network

**Fig. 4:**
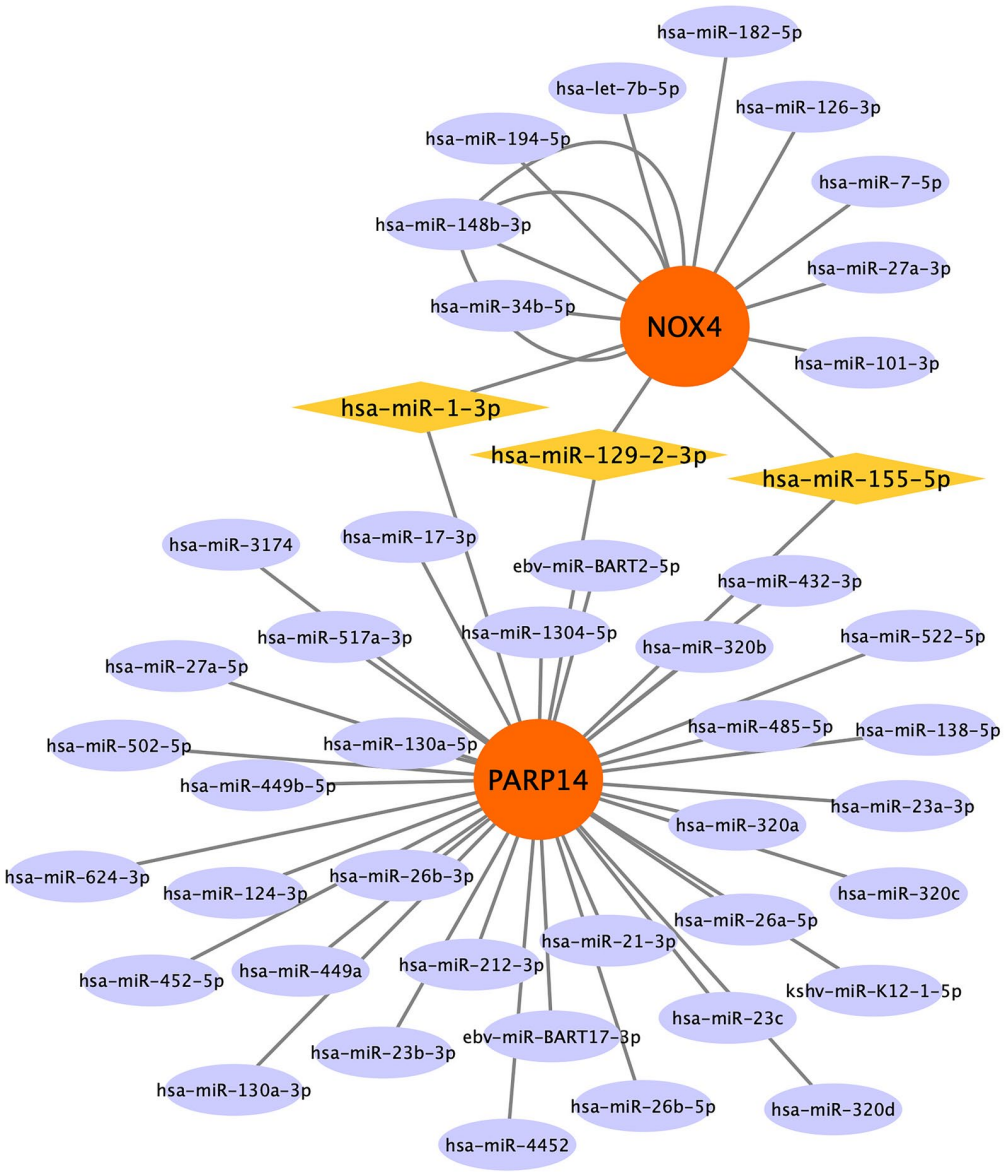
The mRNA-miRNA network. Orange color denotes target genes and yellow color denotes miRNA. Yellow color shows the common miRNA

To accurately identify target genes for miRNAs, we built a miRNA-mRNA network using data from the TarBase V8.0 database and visualized the network through Cytoscape. As shown in Fig. 4, from the results, we got 12 miRNAs linked to the target gene NOX4, and 38 miRNAs linked to the target gene PARP14. Three common miRNAs (hsa-miR-1-3p, hsa-miR-129-2-3p, and hsa-miR-155-5p) were observed in the network.

### Functional enrichment analysis of key genes in DR and AS

**Fig. 5:**
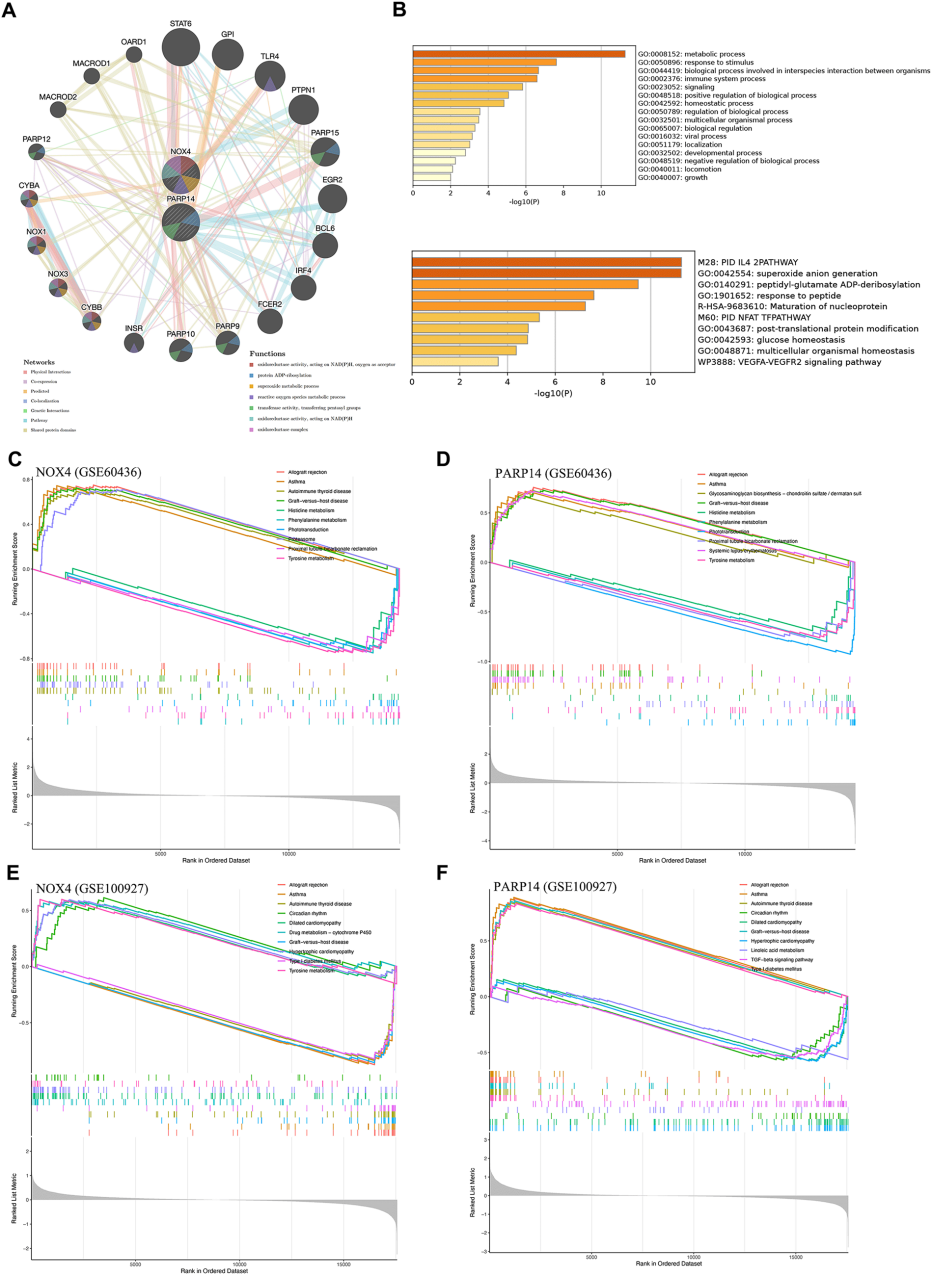
Functional Enrichment Analysis of key genes. **A** Gene interaction network was analyzed with GeneMANIA. The inner circle represents key genes, the outer circle represents genes that interacts with both key genes. **B** Significantly enriched GO terms and KEGG pathways of key genes. Heatmap colored by P-values. **C**–**F** GSEA of NOX4 and PARP14 in DR and AS

The gene–gene interaction network and significantly enriched biological processes of NOX4 and PARP14 were identified through GeneMANIA. The significantly enriched biological processes included oxidoreductase activity, protein ADP-ribosylation, superoxide metabolic process, reactive oxygen species metabolic process, etc. (Fig. 5A). The Metascape analysis displayed the top clusters of enriched sets (Fig. 5B). The key genes showed enrichment in metabolic process, biological process involving in interspecies interaction between organisms, immune system process, etc. The pathway data were enriched in the PID pathway and VEGFA-VEGFR2 signaling pathway, etc. GSEA associated with two key genes was subsequently conducted to compare the different pathways between the disease group and controls. NOX4 and PARP14 were significantly enriched in histidine metabolism, phenylalanine metabolism, and phototransduction in DR (Fig. 5C, D), while significantly enriched in circadian rhythm and dilated cardiomyopathy in AS (Fig. 5E, F). In conclusion, NOX4, PARP14, and their related genes are mainly enriched in oxidative stress, metabolic processes, immune system, and VEGFA-VEGFR2 signaling pathway.

### Immunocyte infiltration and correlation in DR and AS

**Fig. 6:**
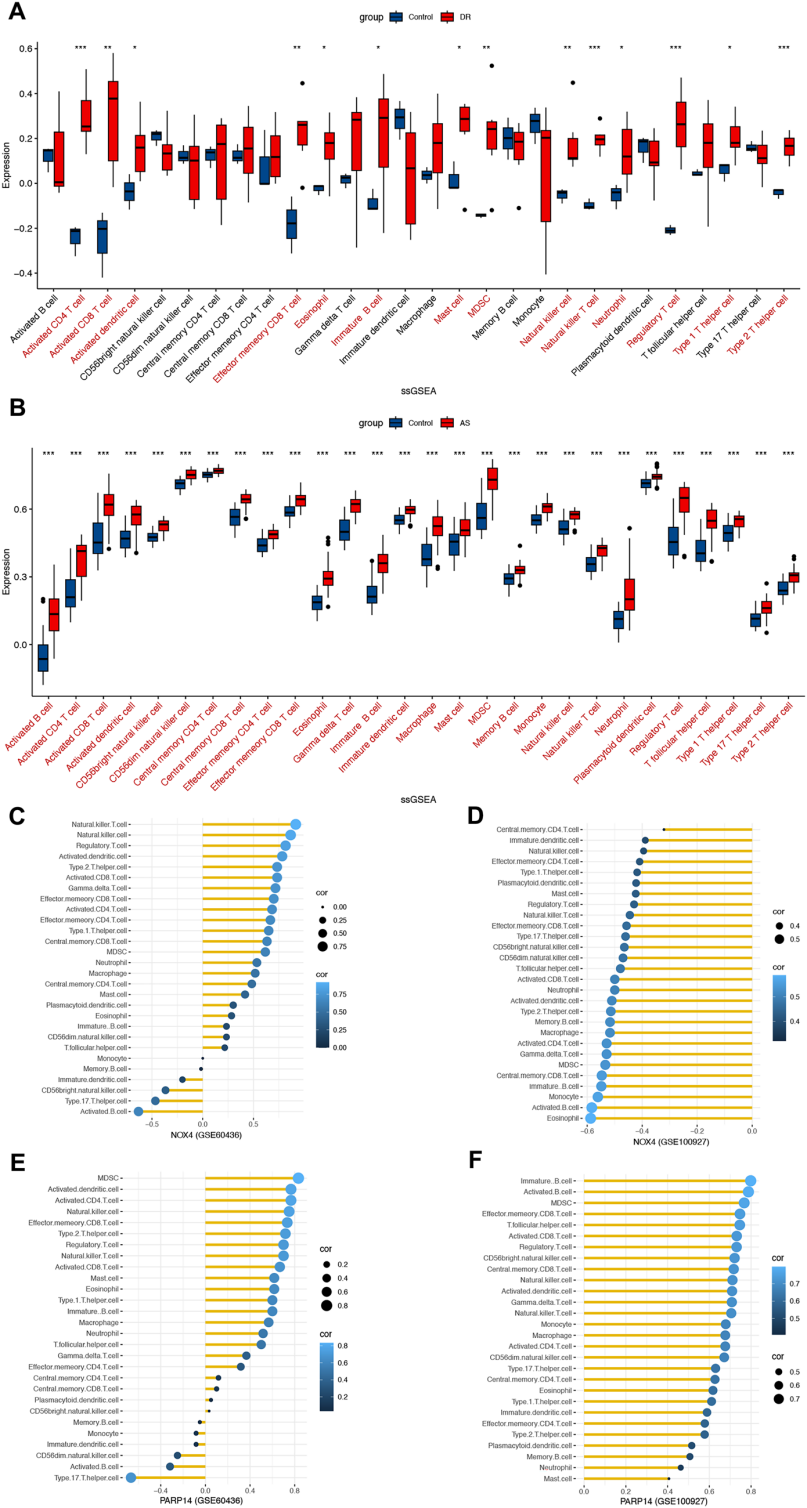
Analysis of immune infiltration in DR and AS. **A** Differential expression of infiltrating immune cells between the disease group and control group in GSE60436. **B** Differential expression of infiltrating immune cells between the disease group and control group in GSE100927. Significant differences are shown in red font. **C**–**F** Correlation analysis between key genes and various immune cells. The more significant the correlation, the larger and the lighter color of the dot. *p < 0.05, **p < 0.01, ***p < 0.001

The CIBERSORT method was applied to evaluate the abundance degree of various immune cells. We further calculate the significant differences in the proportion of immune cells between disease and controls. Differences in activated CD4 T cell, natural killer T cell, T cells regulatory, Type 2 helper T cells (*p* < 0.001), activated CD8 T cell, effector memory CD8 T cells, MDSC, natural killer cell (*p* < 0.01), activated dendritic cell, eosinophil, immature B cell, mast cell, neutrophil, and Type 1 helper T cells (*p* < 0.05) between the DR and control samples were significant (Fig. 6A). Almost all the immune cells expression were significantly different between the AS and control group (Fig. 6B). Correlation analysis between key genes and immune cells was identified using Spearman correlation coefficient, and we visualized the results with lollipop plots. As illustrated in Fig. 6C, the expression of NOX4 demonstrated a strong positive correlation with infiltration abundance of natural killer T cell, natural killer cell, and T cells regulatory, whereas NOX4 expression and activated B cell, Type 17 T helper cell showed a negative correlation in DR. Figure 6E showed that positive correlations were observed between PARP14 expression and MDSC, activated dendritic cell, and activated CD4 T cell, while negatively correlated with Type 17 T helper cell in DR. Figure 6D revealed that there was a negative relationship between NOX4 expression and eosinophil, activated B cell and monocyte in AS. As Fig. 6F shows, the expression of PARP14 was positively correlated with immature B cells, activated B cells, and MDSC in AS. Collectively, activated B cells, MDSC, and Type 17 T helper cells are common immune cells involved in the immune infiltration process of DR and AS.

### Correlation between key genes and ferroptosis marker genes

**Fig. 7:**
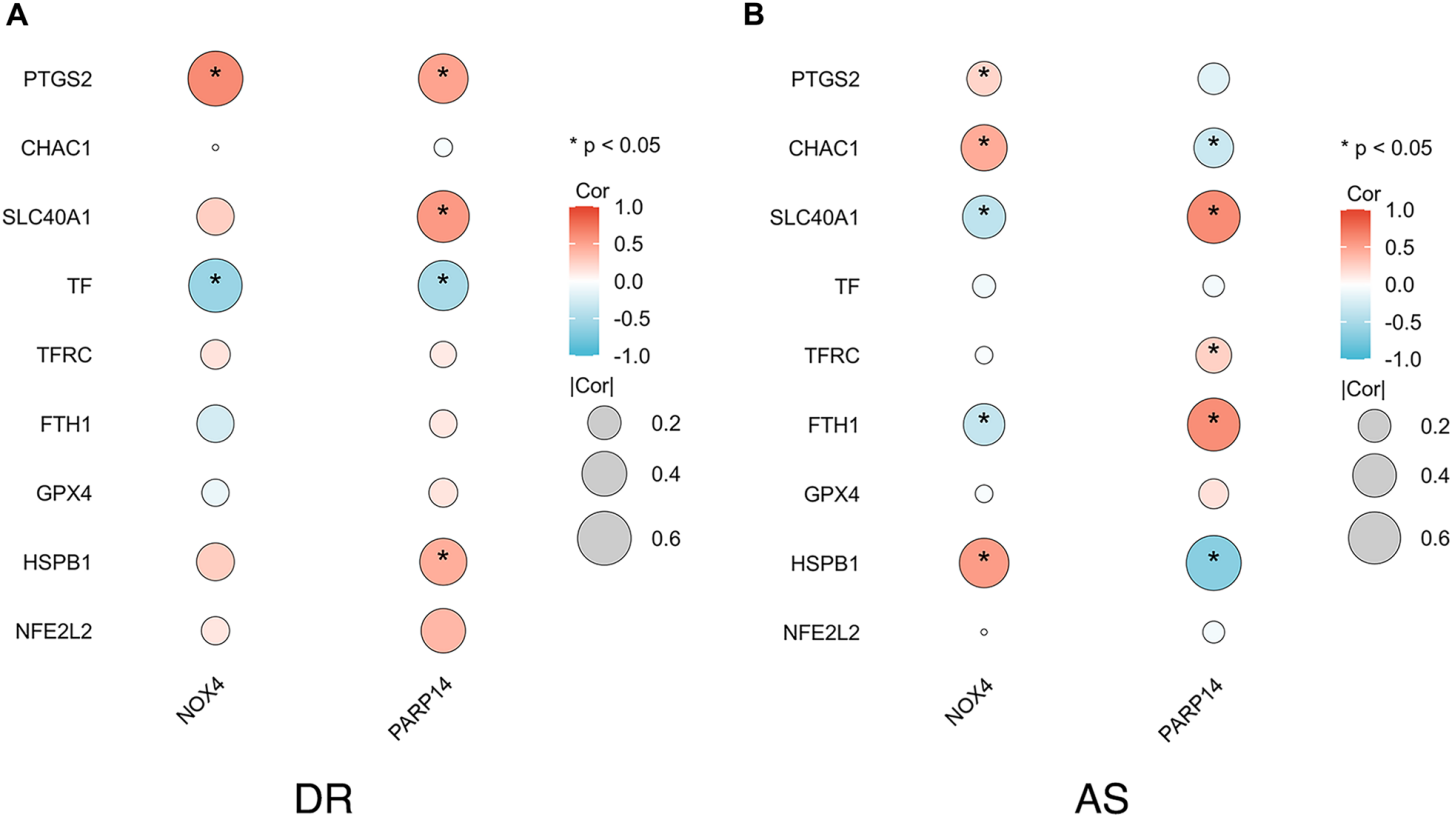
Correlation between key genes and ferroptosis marker genes **A** Correlation between key genes and ferroptosis marker genes in DR.** B** Correlation between key genes and ferroptosis marker genes in AS. The more significant the correlation, the larger the dot size. Red denotes a positive correlation and blue denotes a negative correlation. *p < 0.05

To provide further evidence on the relationship between key genes and ferroptosis, we then downloaded 9 ferroptosis marker genes from the FerrDb database and observed the connection between key genes and ferroptosis marker genes. The results revealed that there are significant correlations between key genes and most ferroptosis marker genes no matter in DR or AS. In all, among these ferroptosis marker genes, PTGS2, TF, CHAC1, SLC40A1, FTH1, HSPB1 are highly correlated with NOX4 and PARP14 (Fig. 7).

## Discussion

This study employed various bioinformatics methods. Firstly, microarray datasets of DR and AS were obtained from GEO, and 307 common DEGs were identified and screened. These were intersected with 484 FRGs extracted from the FERDB database to identify 9 DE-FRGs, including TLR4, PARP12, PARP14, CYBB, NOX4, ABCC5, BID, LIFR, and SNCA. External dataset validation revealed significant differences in NOX4 and PARP14 between disease and control groups of DR and AS, and these two genes showed high diagnostic value. By establishing a miRNA-mRNA network related to NOX4 and PARP14, three miRNA (hsa-miR-1-3p, hsa-miR-129–2-3p, and hsa-miR-155-5p) associated with both diseases were observed. Enrichment analysis showed that key genes were enriched in metabolic processes, biological processes of biointeractions, immune system processes, etc. Pathway data were enriched in the PID pathway, VEGFA-VEGFR2 signaling pathway, etc. Immune infiltration analysis showed that activated B cells, MDSC, and Th17 cells were common immune cells involved in the immune infiltration process of DR and AS. The results of this study contribute to a better understanding of ferroptosis and its role in DR and AS, providing a theoretical basis for immune therapy strategies for DR and AS.

Studies have confirmed the correlation between DR and AS. Retinal microangiogenesis is a major sign of PDR, and angiogenesis is also a common manifestation of progressive AS. The mechanisms of microvascular dysfunction share commonalities with those of large vessel injury. Some biological pathways and mechanisms affect both the development of DR and AS, such as oxidative stress injury mechanisms, chronic inflammation, and growth factors [30]. Ferroptosis is a non-conventional mode of programmed cell death dependent on iron ions. Studies have shown that ferroptosis plays a crucial role in the regulation of diabetes, ocular diseases, and cardiovascular diseases [31]. However, the exact role of ferroptosis in the pathogenesis of DR and AS is not clear, and there is currently a lack of effective treatment strategies. This study identified two key genes related to iron drooping in DR and AS, NOX4 and PARP14.

NOX4 is a regulator of various metabolic processes. In the eye, NOX4 is the main source of ROS in retinal endothelial cells. In the retina of T1D and T2D mice, the expression level of NOX4 is significantly increased. It has also been found to be associated with plaque stability, cell apoptosis, and plaque bleeding in AS [32]. PARP14 (also known as ARTD8 or BAL2) is a member of the PARP family, composed of macro1, macro2, macro3, WWE, and catalytic structural domains. PARP14 uses nicotinamide adenine dinucleotide as a metabolic substrate to mono ADP-ribosylate target proteins, participating in cell responses and immune system signaling pathways. Therefore, PARP14 is considered an attractive target for the treatment of tumors and allergic inflammation [33]. It has been reported that PARP is involved in lipid metabolism and regulates lipid metabolism and homeostasis through transcription factors. The activation of PARP family members is usually a destructive signal of lipid metabolism, and changes in lipid metabolism related to PARP play a core role in the occurrence of cardiovascular aging, atherosclerosis, obesity, fatty liver, hyperlipidemia, skin lesions, type 2 diabetes and its complications [34]. In addition, through enrichment analysis, NOX4, PARP14, and their related genes are mainly enriched in oxidative stress, metabolic processes, the immune system, and the VEGFA-VEGFR2 signaling pathway, etc. Studies have confirmed that destructive oxidative stress is one of the important mechanisms for the occurrence and development of DR and AS [35, 36].

This study observed three miRNAs (hsa-miR-1-3p, hsa-miR-129–2-3p, and hsa-miR-155-5p) related to both NOX4 and PARP14 through the establishment of a miRNA-mRNA network. It has been reported that miRNA plays a core role in cell proliferation, cell apoptosis, and angiogenesis in DR and AS. Researchers have found that hsa-miR-1-3p is a key inhibitor of the occurrence and deterioration of various human malignant tumors [37, 38]. Recent studies have found that hsa-miR-129–2-3p can be used as a biomarker for predicting the severity of DR and early diagnosis of DR [39]. Overexpression of miR-129-2-3p can regulate inflammation and cell apoptosis, promoting wound healing in diabetic patients [40]. However, there are no reports in AS. It has been reported that hsa-miR-155-5p is a medium connecting cellular aging and vascular aging, and its expression level in hVSMCs of AS patients is significantly down-regulated. These findings suggest that hsa-miR-155-5p plays a crucial role in cellular aging [41]. In addition, hsa-miR-155-5p is involved in the pathogenesis of diabetic nephropathy, diabetic neuropathy, and other diabetic complications, but there are few reports in DR. The enrichment analysis results of this study also show that NOX4, PARP14, and their related genes are enriched in the immune system. Our study found that activated B cells, MDSC, and Th17 cells are common immune cells involved in the immune infiltration process of DR and AS. Previous studies have shown that various activities of immune cells such as CD4 T cells, B cells, macrophages, etc., are involved in the pathogenesis of DR and AS [42, 43].

Identifying disease biomarkers through bioinformatics is a common and effective method. Sun et al. [44] used bioinformatics analysis to mine PDR-related genes and pathways. They identified a pathway and eight core genes associated with PDR. The main pathway involved is the cytokines-cytokines receptors interaction, and the core genes are tumor necrosis factor (TNF), tumor necrosis factor receptor superfamily member 12A (TNFRSF12A), C–C chemokine 20 (CCL20), chemokine (C–X–C motif) ligand 2 (CXCL2), oncostatin M (OSM), interleukin 10 (IL10), interleukin 15 (IL15), and interleukin 1B (IL1B). Zhao et al. [45] used bioinformatics methods to explore the gene expression changes in the retina of streptozotocin-induced diabetic rats, obtaining six differential genes. Our study focused on genes related to ferroptosis in DR and AS, so the core genes and pathways were not consistent with the above studies. A common approach is to analyze the correlation between two diseases through comprehensive bioinformatics analysis. Mo et al. [46] discussed the molecular mechanism between osteoporosis and AS. This study is similar to our research method, including identifying DEGs through the GEO database, performing enrichment analysis through GO and KEGG, and constructing a miRNA-gene network. The same method was also applied to the relationship between vascular dementia (VaD) and AS and the evaluation of potential biomarkers. The study identified seven hub genes and screened out two miRNAs significantly associated with AS and VaD [47]. Our study used the above methods to identify ferroptosis-related biomarkers in DR and AS, aiming to analyze the role of ferroptosis in the occurrence and development of DR and AS and to explore related targets. So far, there have been few reports on this topic. This study has certain innovations. The results of this study provide a new perspective on the common molecular mechanism between DR and AS. These common pathways and hub genes may provide promising clues for further experimental research.

Of course, this study inevitably has some limitations. First, only two datasets were included in this study for DR and AS, ignoring the impact of population heterogeneity in different countries on the results. Second, this study is a secondary analysis of existing data, lacking experimental and clinical data validation. Further research includes testing the role of two key genes through animal experiments and collecting clinical samples.

## Conclusion

This study provided an in-depth understanding of the underlying pathogenesis related to ferroptosis and identified new biomarkers NOX4 and PARP14 for DR and AS, the presence of validated biomarkers may provide novel avenues for the early diagnosis of the two diseases in the future.

## Data availability statement

Publicly available datasets were utilized in this study. The data used to support the results of this study are available from the online website mentioned above, and further inquiries can be directed to the corresponding author.

## Data Availability

Data will be made available on request.
